# Sero-surveillance of SARS-CoV-2 specific antibody (IgG) among garment workers in Bangladesh

**DOI:** 10.1038/s41598-025-01183-z

**Published:** 2025-11-25

**Authors:** Nafisa Mosaddek, Hrishik Iqbal, Fatima Farhana, Md. Siddiqul Islam, K. M. Nazmul Hossain, Md. Saiful Islam, K. M. Rakibul Hasan, Abu Syed Md. Mosaddek

**Affiliations:** 1 Bangladesh University of Professionals, Dhaka, Bangladesh; 2 Brac University, Dhaka, Bangladesh; 3 Uttara Adhunik Medical College, Dhaka, Bangladesh; 4 Southeast University, Dhaka, Bangladesh; 5 Quest Bangladesh, Dhaka, Bangladesh; 6 Jahangirnagar University, Savar, Bangladesh; 7 Consultant & Child Development Therapist Paediatric Neurocare & Research (PNR), Dhaka, Bangladesh

**Keywords:** Sero-surveillance, SARS-CoV-2, Antibody, Garment workers, Bangladesh, Health care, Medical research

## Abstract

Since the first detected COVID-19 case in Bangladesh on March 8, 2020, the virus spread rapidly across the country. Despite challenges such as limited healthcare facilities, diagnostic resources, and public awareness, the prevalence of severe symptomatic COVID-19 infections among Bangladeshi working populations, including garment workers, has remained relatively low. This study aimed to determine the sero-prevalence of SARS-CoV-2-specific IgG antibodies among Bangladeshi garment workers. A cross-sectional observational study was conducted on 402 garment workers (69.4% female; mean age = 28.9 ± 6.9 years) in Dhaka. A semi-structured questionnaire was used to collect demographic and COVID-19-related data. Blood samples were analyzed for SARS-CoV-2-specific IgG using a Chemiluminescent Immunoassay. The seroprevalence of SARS-CoV-2-specific IgG antibodies among garment workers was 80.8% (95% CI: 77.1–84.3). No significant differences were observed based on sex, age, marital status, residence, or economic status. Seropositivity was higher among those with RT-PCR-confirmed COVID-19 (96.4%) compared to those without confirmation (79.7%), though the difference was not statistically significant (*p* = 0.054). A significant association was found between comorbid conditions (hypertension, diabetes, asthma) and lower seropositivity (53.3% vs. 81.9%, *p* = 0.015). No significant differences were observed in seropositivity across age, education, marital status, residence, or economic status. High seroprevalence suggests widespread exposure among garment workers, potentially due to occupational factors or preexisting immunity from prior coronavirus infections. Future studies should assess long-term immunity and its implications.

## Introduction

SARS-CoV-2, the causative agent of COVID-19, was first identified in Wuhan, China, in December 2019^1^. This virus, belonging to the Betacoronavirus genus, shares 96% of its genome with the BatCoV RaTG13 strain^2^, and exhibits close genetic similarities to earlier coronaviruses such as SARS-CoV and MERS-CoV. In 2003, SARS-CoV triggered a severe acute respiratory syndrome (SARS) outbreak in Asia, infecting over 8,000 individuals with an approximate 10% mortality rate^3^. Similarly, in September 2012, a novel coronavirus strain, MERS-CoV, was detected in Jeddah, Saudi Arabia^4^. On March 11, 2020, the World Health Organization (WHO) officially declared COVID-19 a global pandemic, given the virus’s rapid international spread^5–8^.

The first confirmed COVID-19 case in Bangladesh was reported on March 8, 2020, after which the virus disseminated rapidly across the country^9^. Serosurveys have become pivotal in assessing population exposure, vaccine coverage, and immunity gaps. Conducting population-based surveillance facilitates tracking infection trends, identifying demographic risk factors, and mapping the geographical distribution of SARS-CoV-2^5–8^.

Multiple countries implemented various serologic testing strategies to monitor SARS-CoV-2 exposure. Finland and the United States utilized rapid diagnostic tests; in the United States, lateral flow immunoassays were commonly applied, while France and Germany relied extensively on ELISA-based methods^10–16^. Community-based testing strategies included drive-through testing in the United States, school-based programs in France, testing in retirement homes in Sweden, representative community screenings in Germany, volunteer screenings in Italy, and broader community-wide testing initiatives in the United States^10–14,17–21^.

The seroprevalence findings from these efforts demonstrated significant variability, ranging from 0.5% in San Miguel County to 14% in Gangelt, Germany, and New York, USA^14,19,21^. Notably, New York’s representative study tested over 3,000 individuals across 40 sites within 19 counties, offering a comprehensive view of population-level exposure^21^.

In India, several sero-surveillance studies were executed nationwide. Between June 27 and July 10, 2020, a study by the National Center for Disease Control (NCDC) in Delhi identified that 23.48% of the population was seropositive for SARS-CoV-2-specific IgG antibodies using ELISA-based testing^22^. A subsequent survey conducted between August 1 and August 7, 2020, reported an increased seroprevalence of 28.3%^23^, indicating that actual exposure rates significantly exceeded the number of laboratory-confirmed cases based on RT-PCR, CB-NAAT, and antigen testing.

In Bangladesh, a nationwide study conducted between April and October 2020 reported seroprevalence rates of 30.4% for IgG and 39.7% for IgM antibodies^24^. Within Dhaka, IgG seroprevalence was recorded at 35.4% in non-slum areas and an alarming 63.5% in slum neighborhoods. Outside Dhaka, metropolitan areas reported a seroprevalence of 37.5%, while rural regions showed lower levels at 28.7%^24^. These disparities can be attributed to variables such as population density, healthcare access, and adherence to preventive measures, including mask-wearing and social distancing.

Despite resource limitations, Bangladesh’s working population, notably garment sector employees, exhibited relatively low rates of severe symptomatic COVID-19 cases. Contributing factors may include the younger demographic of the workforce, leading to milder disease manifestations, and possible development of protective immunity following asymptomatic or mild infections. Public health measures also likely played a significant role in reducing overall transmission rates.

The garment industry is a cornerstone of Bangladesh’s economy, generating approximately $5 billion annually in merchandise exports and employing over 3 million individuals, 90% of whom are women^25–31^. Due to high-density working conditions, the sector is inherently susceptible to SARS-CoV-2 transmission risks. However, until recently, no systematic investigations had been conducted to evaluate seroprevalence or immunity among garment workers.

This sero-surveillance study, therefore, provides critical scientific evidence to inform decision-makers at the Bangladesh Garments Manufacturer and Export Association (BGMEA), government stakeholders, and public health authorities. The findings support the development of robust infection control protocols, vaccination optimization strategies, and effective workforce management practices aimed at safeguarding employee health and sustaining economic resilience.

Accordingly, a population-based sero-surveillance study was initiated to assess the prevalence of SARS-CoV-2-specific IgG antibodies among garment workers, offering essential insights into occupational exposure, immunity patterns, and informing future pandemic response strategies.

### Objectives

The primary objective of this study was to assess the seroprevalence of SARS-CoV-2-specific IgG antibodies among unvaccinated garment workers in garments companies in Bangladesh.

Secondary objectives included:

- To examine the relationship between demographic factors (age, gender, marital status, economic status, education level) and SARS-CoV-2 seroprevalence among garment workers.
- To assess the impact of clinical factors (such as comorbid conditions like hypertension, diabetes, and asthma) on the formation of SARS-CoV-2-specific IgG antibodies.
- To explore the potential role of occupational exposure, considering the crowded working environment of garment factories, and its association with seroprevalence among workers.
- To determine the association between prior symptomatic and asymptomatic COVID-19 infections, confirmed by RT-PCR, and antibody levels in the study population.
- To evaluate whether pre-existing immunity from previous infections contributes to higher seroprevalence and protection against severe disease.

## Methodology

### Study design

This cross-sectional study was conducted among garment workers in Dhaka, Bangladesh, from July to December 2021 in three garment industries. Three garment companies were selected from Dhaka based on their size, geographic location, and willingness to participate in the study. The companies were chosen to reflect a range of working conditions, from large-scale operations to mid-sized ones. Workers within each company were selected using a stratified random sampling method, ensuring representation across various age groups, job types, and gender categories. Strata were defined by these variables, and participants were randomly sampled proportionally within each stratum to reflect the actual workforce distribution. This approach ensures representativeness and minimizes sampling bias across demographic and occupational subgroups.

The National Research Ethics Committee (NREC), Bangladesh Medical Research Council (BMRC), approved this study (Approval No: 2020–2021/306(1–28); Date: 28/06/2021). Signed informed consent was obtained from all participants in accordance with the Declaration of Helsinki. All methods were performed following the ethical guidelines and regulations set by the BMRC.

A total of 402 participants (18–65 years old) were included based on specific inclusion criteria. Blood samples were used as biospecimens to measure SARS-CoV-2-specific IgG antibodies.

The socio-demographic information (e.g., age, sex, residence, employment history, and health status) and COVID-19-related history were collected from participants using a semi-structured questionnaire. The eligibility criteria for participation in the study were as follows:

- **Inclusion Criteria**:
  1. Both male and female unvaccinated workers.
  2. Non-pregnant individuals.
  3. At least two years of uninterrupted employment in the garment industry.
  4. Asymptomatic healthy workers and workers with a history of COVID-19 infection.
  5. Willingness to participate in the study and provide informed consent.
- **Exclusion Criteria**:
  1. Pregnant women, to avoid potential confounders related to altered immune responses during pregnancy.

#### Definition of close contact

Close contact was defined as prolonged exposure (≥ 15 min) within 1 m of a confirmed COVID-19 case within the past 14 days. This criterion is consistent with the guidelines provided by the World Health Organization (WHO) and the U.S. Centers for Disease Control and Prevention (CDC), which define close contact in similar terms. According to the CDC and WHO, exposure to an infected person for at least 15 min within a 1-meter distance significantly increases the likelihood of transmission, especially in enclosed settings where the virus can spread more easily through respiratory droplets.

Blood samples were collected from all participants to measure SARS-CoV-2-specific IgG antibodies. After enrollment, a comprehensive medical history was obtained, and a thorough physical examination was conducted, with all findings documented. No participants were withdrawn from the study. Each participant was assigned a unique sample identification number to ensure anonymity and confidentiality. The study information was strictly protected to maintain participant privacy.

Socioeconomic status (SES) was classified based on monthly income and living conditions, dividing participants into low and middle SES groups. Additionally, confirmed COVID-19 infection was determined through RT-PCR testing, where past infections within the 6 months prior to the study were considered. These classifications and definitions were employed to assess the various factors influencing the study’s outcomes.

### Sample collection procedures

After obtaining informed consent, blood samples were collected using aseptic techniques to prevent contamination. The samples were stored in pre-labeled sterile tubes and immediately placed in dry ice boxes. They were then transported under cold chain conditions to the Medical Center of Jahangirnagar University, Savar, Dhaka, Bangladesh, for SARS-CoV-2-specific IgG antibody assessment.

### Laboratory procedures

Serum levels of SARS-CoV-2-specific IgG antibodies were assessed using the Chemiluminescent Immunoassay (CLIA) technique, following the manufacturer’s instructions (New Industries Biochemical Engineering Co. Ltd, China).

### Sample size determination and power analysis

To ensure statistical validity, a power analysis was conducted to determine the minimum required sample size for estimating SARS-CoV-2-specific IgG seroprevalence among garment workers in Bangladesh. Based on prior serosurveys, the expected seroprevalence (p) was assumed to range between 30% and 60%. Using a 95% confidence level (Z = 1.96) and a 5% margin of error (d = 0.05), the required sample size was calculated using the standard formula for estimating proportions in large populations. The analysis indicated that a minimum of 323 participants was required for 30% seroprevalence, and 369 participants for 60% seroprevalence. Given that this study included 402 participants, the sample size was sufficient to provide statistically meaningful seroprevalence estimates with adequate precision.

### Statistical analysis

Data analysis was performed using SPSS software. Categorical variables were expressed as frequencies and percentages, while continuous variables were represented as mean ± standard deviation (SD). The Chi-Square test was used to assess associations between categorical variables, with p-values < 0.05 considered statistically significant.

## Results

Our study analyzed the seroprevalence of SARS-CoV-2-specific IgG antibodies among garment workers in Bangladesh and no significant differences were observed in socio-demographic characteristics such as age, education, marital status, residence, and economic status (Table 1).

**Table 1:** General characteristics of participants (*n* = 402).

Variables	Categories	*n* (%)
Age (in years) [Mean ± SD]	28.9 ± 6.9	
Sex	Male	123 (30.6)
	Female	279 (69.4)
Level of education	Illiterate	40 (10.0)
	Primary	91 (22.6)
	Secondary	206 (51.2)
	Higher secondary	40 (10.0)
	Graduate	25 (6.2)
Marital Status	Unmarried	56 (13.9)
	Married	346 (86.1)
Residence	Rural	12 (3.0)
	Urban	390 (97.0)
Economic Status	Lower SES	196 (48.8)
	Middle SES	206 (51.2)
H/O close contact with COVID-19 infected patients	Yes	18 (4.5)
	No	384 (95.5)
H/O suffering from COVID-19 infection confirmed by RT-PCR test	Yes	28 (7.0)
	No	374 (93.0)
Suspected sign-symptoms of COVID-19 infection	Yes	122 (30.3)
	No	280 (69.7)
Treatment taken	Yes	120 (29.9)
	No	282 (70.1)
Medications for COVID-19 infection	Yes	88 (21.9)
	Not able mention	31 (7.7)
	No	283 (70.4)
Presence of comorbid conditions (Hypertension, Diabetes mellitus, Asthma)	Yes	15 (3.7)
	No	387 (96.3)

N.P: SES = Socioeconomic Status.

In our study only 28 (7%) of participants confirmed by RT-PCR, study subjects who were suffered from suspected sign-symptoms were 122 (30.3%) whereas only 88 (21.9%) participants had taken the treatment of covid-19 infection and presence of co-morbid conditions in participants (Hypertension, Diabetes mellitus, Asthma) were 3.7%.

**Table 2:** Distribution of suspected sign and symptoms (*n* = 122).

Variables	*n*	(%)
Low grade fever	43	35.2
Dry cough	44	36.1
Fatigue	41	33.6
Sore throat	32	26.2
Headache	75	61.5
Loss of taste and smell	26	21.3
Fever (100–102 °F)	30	24.6
Chill with repeated shaking	9	7.4
Body ache	54	44.3
Muscle pain	60	49.2
Shortness of breath	55	45.1
Chest discomfort	35	28.7

N.P: Each participant may report more than one sign-symptoms.

Most prevalent sign-symptoms of participants who suffered from suspected sign-symptoms of COVID-19 infection but no confirmed by RT-PCR was headache (61.5%) followed by muscle pain (49.2%), shortness of breath (45.1%), body ache (44.3%), dry cough (36.1%), low-grade fever (35.2%) and fatigue (33.6%) (Table 2).

**Table 3:** Distribution of the SARS-CoV-2-specific IgG antibodies with all examined variables (*n* = 402).

Variable	Category	SARS-CoV-2 IgG Positive *n* (%)	SARS-CoV-2 IgG Negative *n* (%)	*p*-value
Sex	Male	96 (78.0)	27 (22.0)	0.419
	Female	229 (82.1)	50 (17.9)	
Marital Status	Unmarried	50 (89.3)	6 (10.7)	0.122
	Married	275 (79.5)	71 (20.5)	
Residence	Rural	10 (83.3)	2 (16.7)	1.000
	Urban	315 (80.8)	75 (19.2)	
Economic Status	Lower SES	162 (82.7)	34 (17.3)	0.440
	Middle SES	163 (79.1)	43 (20.9)	
Close Contact with COVID-19	Yes	14 (77.8)	4 (22.2)	0.974
	No	311 (81.0)	73 (19.0)	
Confirmed COVID-19 by RT-PCR	Yes	27 (96.4)	1 (3.6)	0.054
	No	298 (79.7)	76 (20.3)	
Suspected Symptoms of COVID-19	Yes	100 (82.0)	22 (18.0)	0.811
	No	225 (80.4)	55 (19.6)	
Treatment Taken	Yes	99 (82.5)	21 (17.5)	0.681
	No	226 (80.1)	56 (19.9)	
Comorbid Conditions	Yes	8 (53.3)	7 (46.7)	0.015
	No	317 (81.9)	70 (18.1)	

N.P: SES = Socioeconomic Status.

The overall seroprevalence of SARS-CoV-2-specific IgG antibody was 80.8% among the study participants. No significant differences were observed in seropositivity based on demographic factors such as sex (*p* = 0.419), age (*p* = 0.312), marital status (*p* = 0.122), residence (*p* = 1.000), or economic status (*p* = 0.440). These results suggest that seroprevalence was not significantly influenced by these demographic variables (Table 3).

Among the 28 participants with confirmed COVID-19 via RT-PCR, 96.4% (27/28) were seropositive, compared to 79.7% (298/374) among those without RT-PCR-confirmed infection. This difference was borderline significant (*p* = 0.054), indicating a potential trend towards higher seropositivity among previously diagnosed individuals. The marginal significance observed in our study may be attributed to the relatively small number of RT-PCR-confirmed cases, which reduced the power of the statistical test. Additionally, variations in the timing between the RT-PCR test and antibody test, or potential false negatives in the RT-PCR testing, could also contribute to this borderline result. Additionally, suspected symptomatic individuals had an 82.0% seropositivity rate compared to 80.4% among asymptomatic individuals (*p* = 0.811), suggesting no strong correlation between symptoms and antibody presence.

A significant association was found between the presence of comorbid conditions (hypertension, diabetes, asthma) and SARS-CoV-2 IgG seropositivity. Participants with comorbidities had a 53.3% seropositivity rate compared to 81.9% among those without comorbidities (*p* = 0.015), suggesting that individuals with underlying health conditions might have different immune responses or seroconversion rates.

## Discussion

SARS-CoV-2 has significantly impacted global health and economic conditions, with substantial morbidity and mortality worldwide^32^. Early detection and surveillance remain critical for controlling its spread^33^. Public health experts have emphasized strategies to enhance individual immune responses, aiming to mitigate illness severity, protect against emerging coronavirus strains, and prevent reinfection^34^. Seroprevalence studies provide valuable insights into the extent of population exposure, aiding in the evaluation of public health strategies, vaccination programs, and workplace safety protocols.

Our study observed a high seroprevalence (80.8%) of SARS-CoV-2-specific IgG antibodies among unvaccinated garment workers in Bangladesh. This finding suggests substantial exposure to the virus, likely due to occupational and community transmission. These results are consistent with earlier serological investigations, reinforcing the need for continuous monitoring among high-risk groups^24^. Given that vaccination had not yet been implemented during the study period, it is likely that the antibodies detected resulted from natural infections.

Serological tests detect humoral immune responses and serve as indirect markers of past infection rather than indicators of current disease incidence^35^. Antibodies to SARS-CoV-2 suggest prior exposure; however, their presence does not necessarily confirm long-term immunity or protection from reinfection^36^. It is now well-established that individuals, even after infection or vaccination, can experience reinfections, complicating assumptions regarding herd immunity^37^. Herd immunity thresholds for SARS-CoV-2 were initially estimated to be achieved when approximately 70% of the population had been infected or vaccinated^35^. Nevertheless, the emergence of new variants and waning immunity challenge this concept and highlight the ongoing need for vaccination and booster strategies.

Our findings further underscore the importance of public health interventions, including vaccinations, booster doses, and non-pharmaceutical preventive measures, to mitigate reinfection risks^36,37^. Sero-prevalence analysis across large groups and timeframes remains a critical tool for understanding disease transmission dynamics and informing vaccine design and deployment strategies^37^.

A significant association was found between the presence of comorbid conditions (hypertension, diabetes, asthma) and seropositivity (*p* = 0.015). Individuals with comorbidities exhibited lower seroprevalence (53.3%) compared to those without (81.9%), suggesting variations in immune response dynamics^38–40^. Chronic conditions such as diabetes and hypertension are known to impair immune function, potentially reducing the body’s ability to mount a robust antibody response^39,40^. Additionally, stricter adherence to preventive health measures among individuals with comorbidities may have contributed to reduced exposure and lower seroprevalence. Previous studies have similarly indicated lower antibody responses among individuals with underlying health conditions^38^.

The seroprevalence rate of 80.8% observed in our study aligns with sero-surveillance data reported during earlier phases of the pandemic, wherein antibodies generally develop approximately 1–3 weeks following infection^41^. For example, between April and October 2020, a study in Bangladesh observed IgG seroprevalence rates ranging from 30.4% to as high as 63.5% in certain slum populations^24^. Similarly, a nationwide survey in India conducted between May and June 2020 reported a seroprevalence of only 0.73%^41^, whereas a sero-surveillance conducted in Ahmedabad city between June and July 2020 revealed a crude seropositivity of 17.61% among healthcare workers^42^. In Delhi, between June 27 and July 10, 2020, the National Center for Disease Control reported a seroprevalence of 23.48%^22^. Other studies, such as Minaksi et al., found a seroprevalence of 43.8% with a slight male predominance^43^. In contrast, in our study, females outnumbered males among seropositive individuals, further emphasizing variations across different demographics and settings.

While no statistically significant differences in seropositivity were observed based on demographic factors such as sex (*p* = 0.419), marital status (*p* = 0.122), or economic status (*p* = 0.440), participants with RT-PCR-confirmed COVID-19 exhibited a borderline significant increase in antibody prevalence (*p* = 0.054). This finding reinforces the reliability of serological assays in detecting prior infections. Higher seropositivity among RT-PCR-confirmed cases compared to those without confirmation mirrors previous findings^38^.

It is important to recognize that the seroprevalence captured by antibody testing reflects prior exposure but does not substitute for active case detection. The observed daily fluctuations in positivity rates are driven by symptomatic and asymptomatic infections^32,36^. Therefore, serological testing must be complemented by real-time surveillance to guide epidemiological understanding and resource allocation.

Given that serological tests serve both epidemiological and clinical purposes, they remain crucial for guiding public health policies and tailoring future interventions^33,36^. Future research should prioritize longitudinal cohort studies to assess antibody persistence, reinfection rates, and the long-term effectiveness of immunization strategies^37^.

The findings underscore the necessity of ongoing sero-surveillance to monitor infection trends among high-risk occupational groups. Strengthening workplace safety measures, ensuring timely vaccination, and incorporating regular health monitoring can help mitigate SARS-CoV-2 transmission risks in the garment industry. Future research should expand across multiple industries and geographic locations to enhance the generalizability of these findings.

## Conclusion

The findings of this study indicate a high seroprevalence (80.8%) of SARS-CoV-2-specific IgG antibodies among garment workers in Bangladesh, reflecting significant occupational and community exposure to the virus. The absence of vaccination among study participants further supports the likelihood that immunity was primarily acquired through natural infection rather than vaccine-induced seroconversion.

Statistical analyses showed no significant differences in seropositivity based on demographic factors such as sex (*p* = 0.419), marital status (*p* = 0.122), or economic status (*p* = 0.440). However, participants with RT-PCR-confirmed COVID-19 had a higher seroprevalence (*p* = 0.054), indicating a probable correlation between past infection and antibody presence. Additionally, comorbid conditions significantly influenced seropositivity (*p* = 0.015), suggesting that underlying health factors may affect immune responses.

These findings highlight the importance of continued serological surveillance among garment workers to assess long-term immunity and reinfection risks. Future research should expand on these findings by including a broader range of factories and regions, evaluating antibody persistence, and investigating the effectiveness of workplace interventions in mitigating virus transmission. Strengthening public health policies and workplace safety measures will be essential to protecting this high-risk occupational group from future outbreaks.

### Limitation

Study was conducted only in three industries near Dhaka involving a small number of garment workers thus the sero-prevalence levels should not be generalized among all garment workers of all garment industries in Bangladesh as a whole. Several limitations must be considered when interpreting the findings of this study. First, selection bias could have occurred due to the inclusion of only three garment companies in the Dhaka region, which may not represent the broader garment industry in Bangladesh. Expanding the study to include a larger number of companies from diverse regions would enhance the generalizability of the findings. Second, recall bias may have influenced the accuracy of the self-reported data on symptoms and exposure history, as participants may have had difficulty accurately recalling past events. Future studies could utilize more objective measures, such as medical records, to corroborate self-reported data and minimize this bias.

## Data Availability

The article contains the original contributions discussed in the study. Original datasets are available upon reasonable request from the corresponding author.
