# Correction: Sero-surveillance of SARS-CoV-2 specific antibody (IgG) among garment workers in Bangladesh

**DOI:** 10.1038/s41598-026-36176-z

**Published:** 2026-01-15

**Authors:** Nafisa Mosaddek, Hrishik Iqbal, Fatima Farhana, Md. Siddiqul Islam, K. M. Nazmul Hossain, Md. Saiful Islam, K. M. Rakibul Hasan, Abu Syed Md. Mosaddek

**Affiliations:** 1 Bangladesh University of Professionals, Dhaka, Bangladesh; 2 Brac University, Dhaka, Bangladesh; 3 Uttara Adhunik Medical College, Dhaka, Bangladesh; 4 Southeast University, Dhaka, Bangladesh; 5 Quest Bangladesh, Dhaka, Bangladesh; 6 Jahangirnagar University, Savar, Bangladesh; 7 Consultant & Child Development Therapist Paediatric Neurocare & Research (PNR), Dhaka, Bangladesh

Correction to: *Scientific Reports* 10.1038/s41598-025-01183-z, published online 25 November 2025

The original version of this Article contained an error in the spelling of the author Abu Syed Md. Mosaddek which was incorrectly given as Abu Sayed Md. Mosaddek.

In addition, Abu Syed Md. Mosaddek was incorrectly affiliated with ‘Southeast University, Dhaka, Bangladesh’. The correct affiliations are ‘Uttara Adhunik Medical College, Dhaka, Bangladesh’ and ‘Quest Bangladesh, Dhaka, Bangladesh’.

The original Article has been corrected.

